# Co-prescription of Dual-Antiplatelet Therapy and Proton Pump Inhibitors: Current Guidelines

**DOI:** 10.7759/cureus.21885

**Published:** 2022-02-03

**Authors:** Hannah Saven, Lynna Zhong, Isabel M McFarlane

**Affiliations:** 1 Internal Medicine, State University of New York (SUNY) Downstate College of Medicine, New York, USA; 2 Internal Medicine, State University of New York (SUNY) Downstate Medical Center, New York, USA

**Keywords:** dapt/ppi prescription, cyp450 pathway, upper gastrointestinal bleed (ugib), proton-pump inhibitors (ppi), dual-antiplatelet therapy (dapt)

## Abstract

Dual-antiplatelet therapy (DAPT) prevents thrombotic complications associated with coronary artery disease, acute coronary syndrome, and stent thrombosis following the percutaneous coronary intervention or coronary artery bypass grafting. When initiating DAPT, the risk of thrombosis must be balanced with the increased risk of upper gastrointestinal bleed (UGIB). Proton-pump inhibitors (PPIs) are concurrently prescribed with DAPT to reduce bleeding risk.

In this review, we discuss the benefits and potential complications of DAPT/PPI co-prescription. The only large international randomized control trial (RCT), Clopidogrel and the Optimization of Gastrointestinal Events Trial (COGENT), shows robust evidence that PPIs are a safe and effective method to reduce the risk of bleeding in patients on DAPT. However, more large-scale RCTs are needed to study potential long-term effects and draw a stronger conclusion on this topic.

## Introduction and background

Dual-antiplatelet therapy (DAPT) is the indicated long-term therapy for preventing thrombotic complications associated with coronary artery disease (CAD) and acute coronary syndrome (ACS), and prevention of stent thrombosis following percutaneous coronary intervention (PCI) or coronary artery bypass grafting (CABG) following ST-segment elevation myocardial infarction (STEMI) or non-ST elevation myocardial infarction (NSTEMI) with OR intervention for six months [1,2]. DAPT typically consists of low-dose aspirin in combination with a P2Y12 inhibitor, such as clopidogrel, ticagrelor, or prasugrel, to produce an anti-thrombotic effect. The use of DAPT in treating ACS has led to improved outcomes for patients, especially with the development of more potent P2Y12 inhibitors [3]. Therefore, the increased use of drug-eluting stents for patients with CAD has led to a significant increase in DAPT utilization to prevent stent thrombosis [4]. Stent thrombosis can result in a significant increase in death [4,5]. The administration of DAPT has decreased the risk of stent thrombosis in patients who underwent stent placement [6]. In addition, DAPT has been associated with a reduced risk of myocardial infarction (MI) and stroke [6].

Clinicians have become wary of balancing the bleeding risks associated with DAPT with the risk of stent thrombosis plus other major adverse cardiovascular and cerebrovascular events (MACCE) [7]. Gastrointestinal bleeding, particularly upper gastrointestinal bleed (UGIB), is one of the most common serious bleeding adverse effects of antiplatelet therapy, which has led to the concurrent prescription of proton pump inhibitors (PPIs). However, the use of PPI as gastrointestinal (GI) prophylaxis in the context of DAPT is controversial. This conflicting conclusion and the lack of more randomized control clinical trials (RCTs) have made the safety and efficacy of PPI for GI bleeding prophylaxis for patients on DAPT questionable. In this review, we will discuss the mechanism of DAPT, bleeding risks and epidemiology, and the evidence regarding PPI prophylaxis with concurrent DAPT prescription. This review will consider current guidelines from various medical societies, detail pharmacological mechanisms, and interactions of these medications, and consider studies from the last five years that have built upon the initial data concerning DAPT and PPI co-prescription.

## Review

Methods

We used PubMed to review the literature published between 2016 and 2021 about concurrent DAPT and PPI co-prescription. We chose to review articles from the last five years to limit this review to the most recent literature and keep discussion applicable to current guidelines and practice. The review still included Clopidogrel and the Optimization of Gastrointestinal Events Trial (COGENT), which was published in 2010, because this study is integral to this discussion about DAPT and PPI co-prescription. Searched phrases include “gastrointestinal bleed and antiplatelet therapy,” “gastroprotection and antiplatelet therapy,” “PPI use and antiplatelet therapy,” “DAPT PPI,” and “GI bleeding and CVD.” Duplicate articles were disregarded. Other articles were pulled from an independent search for sections on guidelines, UGIB risks, aspirin, clopidogrel, prasugrel, ticagrelor, and PPI pharmacology and interactions.

Guidelines

The American College of Cardiology (ACC)/American Heart Association (AHA) and European Society of Cardiology (ESC) have published different recommendations for the prescription of DAPT, the duration of treatment, and which agents to prescribe to balance bleeding and ischemic risks.

The 2010 Expert Consensus from the American College of Cardiology Foundation/American College of Gastroenterology/American Heart Association (ACCF/ACG/AHA) stipulates that only patients at high risk of bleeding should receive PPI [7,8]. On the other hand, the 2018 ESC guidelines recommend routine use of PPI for all patients on DAPT [9]. This difference in clinical guidance is largely based on differences in the interpretation of a major clinical trial (COGENT) that demonstrated a pharmacologic interaction between clopidogrel and omeprazole without a clinically significant change in adverse cardiovascular events [7-10]. According to ACCF/ACG/AHA guidelines, patients on DAPT with multiple risk factors for GI bleeding can appropriately be prescribed PPI. The use of PPIs or histamine-2 receptor antagonists (H2RAs) in patients with a low risk of upper GI bleed only showed marginal benefit. Since 2010, no updates have been published to these guidelines [8]. The 2016 ACC/AHA guidelines reiterated this focus on using PPI in only high-risk patients [2].

The ESC recommends that “every effort should be pursued to mitigate the risk of bleeding complications while the patient is on DAPT as appropriate, and routine use of PPI” [9]. The authors cited increased mortality and morbidity associated with bleeding events after successful PCI and, therefore, encouraged the prescription of PPI to all patients on DAPT.

The 2020 International Consensus Recommendations on the Management of Patients With Nonvariceal Upper Gastrointestinal Bleeding state that patients with previous ulcer bleeding receiving cardiovascular prophylaxis with single- or dual-antiplatelet therapy should receive PPI therapy [11]. However, this was a “conditional recommendation” with “low-quality evidence,” due to the lack of RCTs. Additionally, these guidelines considered the potential for increased risk of mortality or MI with concurrent DAPT/PPI prescription. Through their own meta-analysis, these authors did not find an increase in mortality from these events in patients receiving DAPT and PPI [11].

The National Association of Hospital Cardiologists (ANMCO) and the Italian Association of Hospital Gastroenterologists (AIGO) recommend the use of PPIs in patients on one antiplatelet agent if GI risk factors are present [12]. Patients receiving antiplatelets and who have a history of peptic ulcer disease (PUD), concomitant use of another antiplatelet agent, concomitant use of vitamin K-antagonists (VKAs) or direct-acting oral anticoagulants (DOACs), concomitant use of non-steroidal anti-inflammatory drugs (NSAIDs), or concomitant use of steroids should be further considered for PPI use due to increased risk of bleeding. Other risk factors included are age over 65 years, dyspeptic symptoms, and gastroesophageal reflux disease (GERD).

Pharmacology

**Figure 1:**
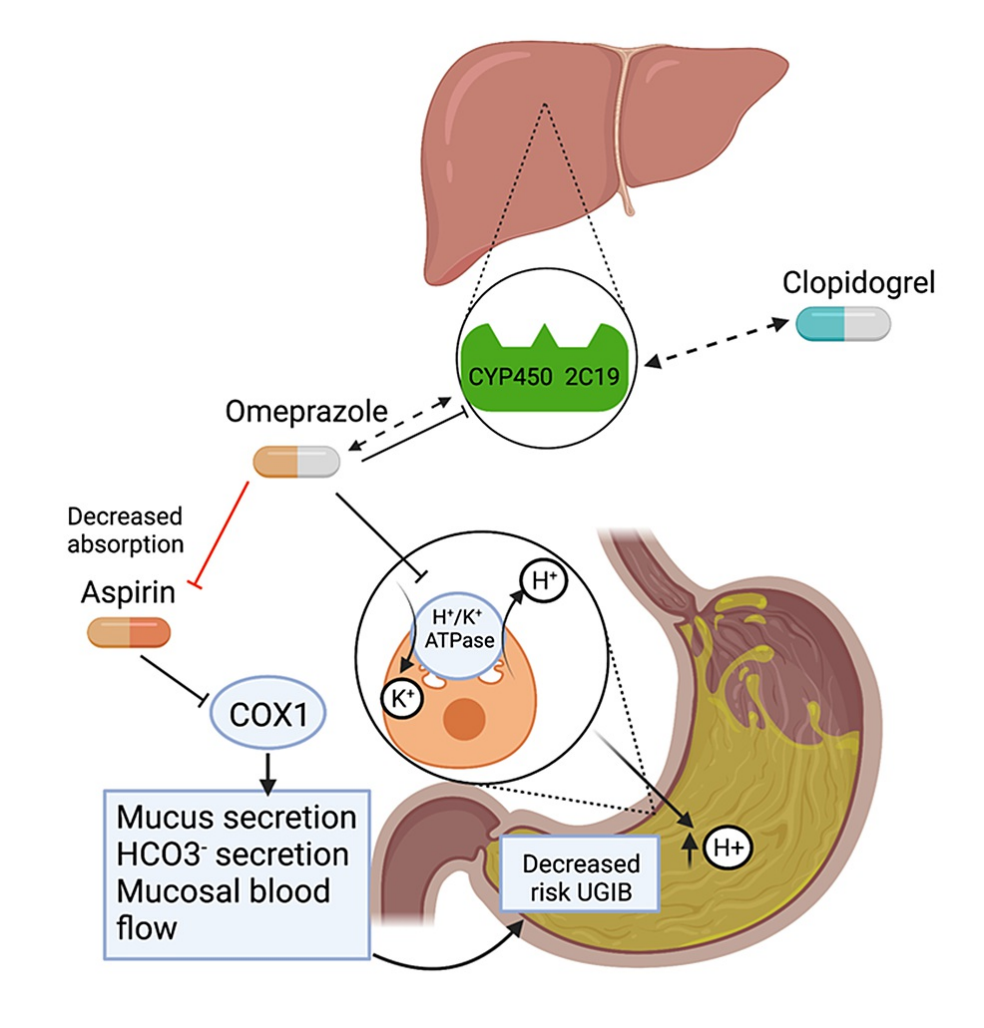
Proposed mechanism for DAPT/PPI drug interaction. Clopidogrel is metabolized by cytochrome CYP450 2C19. Omeprazole has inhibitory effects and is metabolized by CYP450 2C19, therefore, potentially limiting the active form of clopidogrel and its antiplatelet effects. Omeprazole inhibits the H+/K+-ATPase on the luminal side of parietal cells, which decreases the acid production in the stomach. This could limit the absorption of aspirin. On its own, aspirin increases the risk of UGIB by decreasing mucus secretion, decreased bicarbonate secretion, and decreasing mucosal blood flow. The addition of PPI to aspirin typically works to decrease the risk of ulcer formation via decreased acid production. Omeprazole has the potential to affect the absorption and metabolism of aspirin and clopidogrel, respectively. Created with BioRender.com. DAPT, dual-antiplatelet therapy; PPI, proton pump inhibitor; UGIB, upper gastrointestinal bleed; COX1, cyclooxygenase-1; H+/K+-ATPase, hydrogen potassium adenosine triphosphatase.

DAPT includes two pharmacotherapies: aspirin, an NSAID, and a P2Y12 inhibitor, such as clopidogrel, prasugrel, or ticagrelor. Aspirin has a key role in antiplatelet therapy. Its mechanism of action is the irreversible inhibition of the cyclooxygenase-1 (COX1) enzyme in the arachidonic acid pathway to limit inflammation. For its antiplatelet role, the inhibition of COX1 prevents the conversion of arachidonic acid into prostaglandin G2/H2 and subsequent reactions by thromboxane synthase into thromboxane A2 (TXA2) [3]. This mechanism makes aspirin effective in preventing serious vascular adverse effects such as MI and stroke. Conversely, aspirin has been infamously associated with an increase in bleeding even when used as monotherapy [3]. By inhibiting COX1, aspirin depletes the protective prostaglandins that promote the integrity of the stomach lining. COX1 inhibition results in decreased mucus secretion, decreased bicarbonate secretion, and mucosal blood flow (Figure 1) [13]. These processes promote UGIB. In a review for the U.S. Preventive Services Task Force (USPSTF), patients who underwent PCI and received low-dose aspirin had a significant increase in major gastrointestinal bleeding risk due to small intestinal mucosal injury [14,15]. The addition of DAPT therapy did not increase the risk of aspirin-induced small intestinal mucosal injury, although only 10 patients were in the DAPT group [4].

Thienopyridines like clopidogrel or prasugrel work by inhibiting the binding of adenosine diphosphate (ADP) to its P2Y12 ADP receptor and requires the transformation by cytochrome P450 enzymes to be activated [16]. In its active form, clopidogrel binds irreversibly to platelet P2Y12 ADP receptor, therefore, inhibiting platelet aggregation. Studies have shown that clopidogrel may have a suboptimal response that can limit its use as an antiplatelet agent in coronary stenting and ACS [17,18]. Prasugrel is more potent than clopidogrel and does not require as many steps in hepatic activation, making it a preferred agent for preventing thrombosis [3]. The main metabolic difference is an enzyme called human carboxylesterase (hCE), which results in a faster onset, greater magnitude, and more consistent levels of platelet inhibition [3]. However, greater potency of the antiplatelet effect also increases bleeding risk. The Greek Antiplatelet (GRAPE) trial showed that prasugrel-treated patients with ACS undergoing had more frequent bleeding than clopidogrel-treated patients [19].

Ticagrelor is a cyclopentyltriazolopyrimidine (CPTP) that reversibly binds to the P2Y12 receptor and noncompetitively with ADP [3]. Unlike clopidogrel and prasugrel, ticagrelor is not a prodrug and does not require metabolic activation to exert its effects [3]. Compared to clopidogrel, ticagrelor has also demonstrated greater antiplatelet efficacy [20]. One study showed that ticagrelor had more frequent bleeding compared to clopidogrel-treated patients [19]. Conversely, another study showed that ticagrelor is non-inferior to clopidogrel in both major bleeding events without differences in ischemic events [21]. More data concerning ticagrelor bleeding risks are necessary to provide clinical guidance.

When DAPT is used, there is a two-fold effect on platelets: aspirin inhibits the production of TXA2 and clopidogrel inhibits P2Y12, both of which inhibit platelet activation and platelet aggregation. P2Y12 receptor inhibitors are not directly responsible for creating ulcers in the gastric lining as aspirin is, but its antiplatelet activity confers a potential risk for bleeding.

PPIs directly stop gastric acid secretion by irreversibly binding to hydrogen potassium adenosine triphosphatase (H+/K+-ATPase) on the luminal membrane of parietal cells in the lining of the stomach (Figure 1). This group of medications includes omeprazole, lansoprazole, pantoprazole, rabeprazole, esomeprazole, and dexlansoprazole. PPIs are metabolized by and competitively inhibit CYP2C19 (Figure 1) and CYP3A4 pathways except for pantoprazole, lansoprazole, rabeprazole, and dexlansoprazole. According to the American College of Gastroenterology (ACG), the PPI indications include PUD, GERD, erosive esophagitis, and hypersecretory pathologies like Zollinger-Ellison syndrome [22]. PPIs may also be used to prevent stress ulcers in ICU settings and prevent the formation of ulcers from NSAID use. Full adherence to PPIs was the only gastroprotective approach that significantly reduced the risk of UGIB when taking NSAIDs even at low doses, with even greater risk at higher dose NSAIDs [23]. The same effect could not be achieved with histamine 2 receptor antagonists (H2RAs). While PPIs block H+/K+-ATPase on the luminal face of the parietal cells, H2RAs indirectly inhibit acid secretion by binding to the histamine 2 receptor on the basolateral side of the parietal cell. This receptor works in conjunction with acetylcholine and gastrin receptors to promote acid secretion from the parietal cell. However, there is varied evidence as to whether H2RAs are effective in preventing NSAID-induced bleeding or DAPT-induced bleeding. One study showed that long-term PPI use reduced the risk of UGIB recurrence, but this effect was not observed with patients on H2RAs [24]. On the opposite side, no significant GI bleeding was reported in patients at risk for recurrent GI bleeding who were on low-dose aspirin regardless of if they had received rabeprazole [25]. Furthermore, other studies proposed that H2RAs prevented low-dose aspirin-related GI bleeding and ulcer formation significantly less than PPI [26,27].

Over the last few years, clinicians have been wary of prescribing PPI due to observational studies proposing correlations with adverse effects like chronic kidney disease (CKD) and dementia [12]. The ACG acknowledges side effects of PPIs have been reported in retrospective observational studies [22]. However, no RCTs have been performed to assess the validity of these adverse effects [28]. Low-quality evidence should not be used to guide clinicians and withhold PPIs from patients who might benefit from its use [29].

Epidemiology: who is at risk of bleeding?

DAPT can put certain patient groups at increased risk of bleeding. ACCF/ACG/AHA currently recommends PPI prophylaxis for patients on DAPT who have *Helicobacter pylori* infection, advanced age, or use of warfarin, steroids, or NSAIDs [8]. In these individuals, the risk of bleeding outweighs the risk of thrombosis. Other risk factors for an upper GI bleed identified by this study include female sex, major organs dysfunction, hemostatic disorders, or a history of hemorrhage [30].

Older patients tend to develop disabling or fatal UGIBs compared to younger patients [31]. Younger individuals were more likely to have non-disabling UGIB with lower case fatality [3]. Older patients who are at higher risk for UGIB had decreased mortality when prescribed PPI along with DAPT. However, the ceiling effect could also account for an imprecise number needed to treat (NNT) for PPI in individuals aged >85 years. Five-year risk of bleeding can exceed the life expectancy of elderly patients, resulting in artificial inflating NNT [32]. In addition to advanced age and female gender, *H. pylori* infection, history of digestive tract disease, diabetes, hypertension, dyslipidemia, anemia, CKD, peripheral vascular disease, and smoking have been recognized as risk factors for increased risk of GI bleed [33]. Patients in the high-risk bleeding group were also more likely to have had a previous MI, previous PCI with stent implantation, surgical revascularization, and stroke [34].

Who is getting a DAPT/PPI prescription?

Since 2010, concurrent use of clopidogrel and PPI has steadily decreased due to the 2009 FDA warning that omeprazole and esomeprazole could limit the antiplatelet activity of clopidogrel [35]. This finding led to the updating of clopidogrel’s label noting its interaction with omeprazole. Non-inhibiting PPIs such as pantoprazole, lansoprazole, rabeprazole, and dexlansoprazole prescription rates were not affected by this warning. In 2016, the prevalence of clopidogrel and inhibiting PPIs among ACS patients was 0.8% compared to 34.9% in 2009. This study also found that younger inpatients were less likely to receive concurrent treatment compared to older groups, defined as 65 years or older.

Although European guidelines recommend all patients receiving DAPT should take PPI, similar rates of co-prescription are seen between European countries and the United States. When considering ESC guidelines, PPI prescribing trends in patients taking DAPT seven days following hospital discharge for an acute MI did not meet ESC criteria that recommended PPI prescription for all patients on DAPT regardless of upper GI risk factors [9,36]. Only 35% of patients with a higher risk of UGIB received recommended PPI treatment [36]. Overall, prescribing trends are much lower than recommended. This observation is reflective of the overall skepticism of prescribing PPI to patients on DAPT despite guidelines.

DAPT increases bleeding risk

DAPT increases bleeding risk because of the pharmacological interaction with platelet function. Dewilde et al. (2013) studied bleeding complications on PCI patients who used oral anticoagulation (AC) and clopidogrel with or without aspirin [37]. AC increases the risk of bleeding. Patients receiving double therapy (AC and clopidogrel) had fewer bleeding episodes than those on triple therapy (AC, clopidogrel, and aspirin) [37]. Clopidogrel monotherapy in PCI patients was associated with a significantly lower rate of bleeding complications compared to dual therapy (clopidogrel and aspirin). Importantly, there were no additional thrombotic events in the monotherapy group. Adding aspirin to clopidogrel in PCI patients results in an increased bleeding risk [38-40]. Some of these studies suggest aspirin is the component of DAPT that increases the risk for GI bleeding [38,39]. One study has shown a two- to three-fold increased risk of gastrointestinal bleeding and another major bleeding in DAPT compared to monotherapies [39]. However, low-dose aspirin had a better safety profile compared to other pharmaceuticals [39]. In the same study, clopidogrel was associated with a non-significant increased risk of major bleeding events and was found significantly better in secondary prevention than low-dose aspirin in the prevention of event-free survival.

Notable interactions

Aspirin/PPI Interaction

In the context of DAPT, controversy remains as to whether concurrent use of PPI is more harmful or helpful. The acidic nature of the stomach facilitates the absorption of aspirin; therefore, the administration of PPIs decreases the acid secretion and increases gastric pH, which can potentially interfere with aspirin absorption (Figure 1). A case-control study of CAD patients treated with and without PPIs found that platelet aggregation and activation were significantly increased in the patients receiving PPI, increasing the chances of thrombotic events [41]. However, the study does not elucidate the causality of PPI treatment to aspirin response. On the other hand, other researchers argue that concurrent PPI and aspirin use could prevent upper GI toxicity from aspirin therapy, and coadministration would increase adherence to aspirin therapy [42]. Compared to nonuse of PPIs, continuous PPI use was associated with a lower risk of aspirin discontinuation among patients with a high risk for upper GI events [43].

Clopidogrel/PPI

Due to clopidogrel’s metabolism by CYP450 liver enzymes and some PPIs inhibition of CYP450, concurrent use of clopidogrel and PPIs could decrease the overall efficacy of clopidogrel (Figure 1). A study by Juurlink et al. (2009) was one of the first studies to describe this interaction via the CYP450 pathway [44]. In this study of patients following MI, researchers found that the use of PPIs with clopidogrel was associated with a 40% increase in the risk of recurrent MI within 90 days of hospital discharge [44]. However, pantoprazole, which does not inhibit CYP450 2C19, was not associated with readmissions for myocardial infarction.

In contrast, another study demonstrated that PPI and clopidogrel use in patients after ischemic stroke was not associated with recurrent ischemic stroke, MI, intracerebral hemorrhage, and death [45]. These authors concluded that PPIs did not affect platelet aggregation in patients taking clopidogrel. More research is needed to investigate the proposed CYP450-related interactions between concomitant PPI and clopidogrel therapy.

Prasugrel/PPI and Ticagrelor/PPI

With newer P2Y12 inhibitors earning favor over clopidogrel, the use of PPIs may become even more important because these newer agents are more potent. The stronger antiplatelet effect of prasugrel can lead to renewed bleeding concerns. There is less literature concerning interactions between PPIs and prasugrel or ticagrelor. Like clopidogrel, prasugrel is still metabolized by CYP2C19 into its active form [12]. One study found that patients on prasugrel and PPI were less likely to have MI than patients on clopidogrel and PPI [46]. However, this study had a surrogate endpoint, which led to skepticism over the increased observed cardiovascular risk [12]. One position paper from Italy stated that if a patient is on clopidogrel, then rabeprazole or pantoprazole should be the PPI of choice if patients have at least one risk factor [47]. If a patient is on prasugrel or ticagrelor with at least one GI bleeding risk factor, any choice of PPI may be used [47]. Despite these recommendations, there is little evidence to support interaction or lack thereof between prasugrel and PPIs. Conversely, another study found that PPI use in patients did not significantly affect the comparative effectiveness or bleeding risk of prasugrel compared to clopidogrel [48].

In theory, ticagrelor should not hepatically interact with PPIs since it is not a prodrug and does not need to be metabolized. Its stronger potency may lead to increased bleeding risks, though this risk has yet to be thoroughly studied [19-21]. In terms of interactions with PPI, one study that looked at concomitant use of PPI and clopidogrel or ticagrelor in patients with ACS did not find any significant differences between all-cause death, re-infarction, or decreased risk of severe bleeding [49]. A separate study substantiated the lack of difference in death, MI, and GI bleeding between ACS patients treated with clopidogrel or ticagrelor and PPIs [50]. While the current literature is limited, the results indicate that these separate agents do not change major outcomes when taken with PPI and that these results are like those of patients that took clopidogrel and PPI despite pharmacological differences.

Use of PPI while on DAPT

While co-prescription of PPI alongside DAPT is associated with decreased risk of UGIB, it has also been associated with decreased efficacy of clopidogrel in preventing thrombosis. Only one RCT to date has studied co-therapy of DAPT and omeprazole [10]. In the COGENT, patients taking DAPT and omeprazole had significantly less overt or occult bleeding, symptomatic gastroduodenal ulcers or erosions, and obstruction or perforation than patients taking DAPT with no PPIs. Patients taking esomeprazole also had decreased rate of overt UGIB (HR, 0.13; 95% CI, 0.03-0.56; P = 0.001). There were no significant differences between omeprazole and placebo groups in deaths from cardiovascular causes, nonfatal myocardial infarction, revascularization, or stroke (HR with omeprazole, 0.99; 95% CI, 0.68-1.44; P = 0.96). The study controlled for NSAID use and *H. pylori* infection. Unfortunately, the trial lost sponsor financing and was terminated early. Unlike previous observational studies, this RCT concluded that DAPT and PPI, specifically clopidogrel and omeprazole, did not increase the risk for cardiovascular adverse events. The study was limited by decreased power as fewer adverse events occurred than anticipated. Additionally, COGENT excluded patients with prior indications for PPIs/H2RAs as well as patients with upper GI pathologies and previous AC or fibrinolytic therapy. The generalizability of the results of the COGENT study may also be limited as 94% of study participants were White and only one PPI, omeprazole, was used. According to COGENT results, the benefit of DAPT and PPI co-therapy (decreased GI bleeding) outweighed the potential risks (adverse cardiovascular events due to decreased DAPT efficacy).

Post hoc analysis of COGENT patients [51] found that among PCI-treated patients, omeprazole significantly reduced rates of composite gastrointestinal events without increasing composite cardiovascular events. Similarly, in patients with ACS, omeprazole lowered the risk of primary gastrointestinal events compared to placebo without a significant increase in cardiovascular events. This post hoc analysis in high-risk cardiovascular patients supports the use of clopidogrel and PPIs without increasing the risk for cardiovascular complications.

In another post hoc analysis, high-dose and low-dose aspirin had similar risks of gastrointestinal events and major adverse cardiac events aspirin [52]. The addition of PPI therapy led to a reduction of primary GI endpoint in both subsets. These various analyses of COGENT highlight the safety of concurrent PPIs with DAPT therapy and further support its efficacy in decreasing the incidence of upper GI bleeding without major side effects.

Additional studies have corroborated the finding that PPIs reduce the risk of UGIB, GI ulcers, and erosions in patients on concurrent DAPT [36,53-56]. Screening for risk factors of upper GI bleeding did not significantly reduce the incidence of UGIB in patients placed on DAPT and PPI [57]. The ACCF/AGA/AHA only recommends PPIs concurrently with DAPT in groups with bleeding risk factors [8]; the benefit of PPIs and DAPT therapy outweigh any potential risks, but more research is needed to determine whether this benefit may only apply to patients in a high-risk group [36,57].

There is evidence beyond COGENT data that PPI and DAPT co-therapy does not lead to an increase in MACCE. There are substantial data that demonstrate that patients treated with PCI and DAPT (clopidogrel and aspirin) have had similar all-cause death, MI, and cerebrovascular accident (CVA) among PPI and non-PPI users [27,53-60]. A couple of studies in patients with CAD also concluded PPI and DAPT co-prescription did not lead to increased adverse risks [56,60]. Patients screened for UGIB risk factors such as old age, dyspepsia, previous uncomplicated ulcer, previous ulcer bleeding, NSAID, corticosteroid, selective serotonin reuptake inhibitor (SSRI), or oral anticoagulant on DAPT/PPI therapy had significantly fewer events of unstable angina pectoris compared to controls [57]. This development is in line with ACCF/ACG/AHA guidelines [8]. Compared to COGENT, the Prolonging Dual-antiplatelet Treatment after Grading Stent-induced Intimal Hyperplasia Study (PRODIGY) included patients with a higher risk of bleeding or patients that had other indications for PPIs [57]. The patients on PPIs were more likely to be older, female, have lower creatinine clearance, higher bleeding score, and present more frequently with ACS [58]. Despite these increased risk factors for bleeding, patients on PPIs/DAPT and DAPT had similar rates of all-cause death, MI, and CVA. Though these studies point to a favorable safety profile between DAPT and PPI, one study pointed out a higher risk of ischemic stroke and MI observed in long-term PPI use [36]. This concern has already been addressed by the ACG [22]. In the last five years, there has been a myriad of data establishing that PPI does not decrease the efficacy of DAPT.

Some studies propose that the use of PPIs with DAPT may in fact decrease MACCE risk [61]. Patients screened for UGIB risk and prophylactically treated with pantoprazole had increased compliance to DAPT [57,61]. This mirrors the mechanism proposed by the aspirin/PPI co-administration [42]. Patients who were screened and given PPI prophylaxis had higher compliance with treatment of an ADP-receptor inhibitor like clopidogrel [57]. The researchers predicted that dyspepsia related to antiplatelet therapy was reduced by taking PPIs. The use of PPIs to not only prevent DAPT-related bleeding but also to treat dyspepsia symptoms caused by DAPT could be a novel prospect [43].

Potential racial and ethnic differences should also be considered. One meta-analysis/systematic review of observational studies [62] showed that among Caucasian populations, DAPT and PPI concurrent use was associated with an increased risk for MACCE, all-cause death, and all other studied clinical adverse events. However, in Asian populations, no significant differences in MACCE, all-cause death, cardiac death, and stroke were found between PPI and non-PPI users. Without racial stratification, the results of this study did not show any benefit in the concurrent prescription of PPIs and DAPT [62]. This new perspective could elucidate why studies offer contradictory findings of DAPT and PPI concurrent use. When studying stent implantation, one study found that MACCE was increased in PPI users due to increased stent thrombosis, without differences in death, ST, or MI compared with non-PPI users [63]. However, these differences were driven by underlying confounding patient characteristics in the PPI group such as higher incidence of bifurcation lesions, bare-metal stents, and higher risk of target lesion revascularization rather than increased atherothrombotic risk [63]. Study limitations include observational nature, physician discretion for PPI prescription, lack of PPI compliance follow-up, and patient-reported DAPT adherence [63]. Additionally, DAPT compliance was self-reported.

Discussion

The concurrent use of DAPT and PPIs has continued to be debated since COGENT results were published in 2010. Despite its loss of funding and early termination, COGENT had enough data to show that concurrent therapy with PPI and DAPT led to a significant reduction of upper GI bleeds, erosions, and ulcers without increasing adverse events. However, subsequent observational studies suggested that PPIs and P2Y12 inhibitors were associated with increased major cardiac adverse events, via CYP450 inhibition, demonstrating statistically significant results (p = 0.05) with an OR of 1.27 for reinfarction and 1.4 for risk of myocardial infarction [44].

As COGENT has been the only large, multi-center, international, double-blind, phase 3 RCT in this topic, there is a paucity of data needed to yield strong recommendations on DAPT and PPI co-therapy. In the past five years, the academic milieu has been flooded with studies providing murky evidence for proposing PPI protection for GI bleed for patients on DAPT therapy or arguing PPIs decrease the efficacy of DAPT therapy, therefore, increasing the risk of cardiovascular adverse events or stent thrombosis. To build upon COGENT, future studies should include stratification of medications with and without CYP450 inhibition concurrently with DAPT. While there have been studies that look at how UGIB screening risk affects the incidence of bleeding [57], no RCT study has investigated the effects of PPI and DAPT in patients with a history of ulcer bleeding. Additionally, since the COGENT study ended early, new studies should also aim to examine any long-term effects of concurrent therapy.

Another factor to consider is potential racial and ethnic differences; concurrent PPI and DAPT have been studied in majority Caucasian and Asian populations [62]. The authors propose that patients with a hypofunctional CYP2C19 metabolic phenotype may benefit from concurrent DAPT/PPI therapy [62]. Racial and ethnic differences could contribute to the variation in study findings of DAPT and PPI concurrent use. More research is needed to further investigate this mechanism and could potentially provide more personalized care to patients.

Current American clinical guidelines tend to value conservative management, recommending PPI co-prescription with DAPT only in patients with a high risk of bleeding [8]. As it stands, there is not enough evidence to recommend PPI to all patients receiving DAPT [8]. However, the benefits of concurrent DAPT/PPI prescription are not limited to a decrease in UGIB risk. For example, some studies have shown patients on DAPT and PPIs could benefit from increased medication compliance to DAPT [57].

Future studies should also consider the use of third-generation P2Y12 inhibitors such as ticagrelor. Most studies only considered clopidogrel as the antiplatelet agent for DAPT. COGENT only examines the effects of clopidogrel. Comparing the efficacy of these newer agents concurrently with PPIs could provide meaningful data about the best combination of drugs that maximizes efficacy and decreases the risk of adverse effects. For example, ticagrelor, unlike clopidogrel, does not need hepatic enzyme activation [3,64]. Therefore, this could be a viable solution for clinicians and patients concerned with both gastrointestinal bleeding and potential interactions between clopidogrel and a PPI. The current literature on the use of newer, alternate P2Y12 and PPIs is quite limited, and more research is needed to determine if there is any interaction between PPIs and ticagrelor or prasugrel, or to determine if there is any benefit in using these agents concurrently with PPIs instead of clopidogrel. These data will be important especially since ticagrelor and prasugrel are becoming preferred agents over clopidogrel, which has had inconsistent antiplatelet responses [17,18]. Therefore, this could be a viable solution for clinicians and patients concerned with both gastrointestinal bleeding and potential interactions between clopidogrel and a PPI. For many of these studies, particularly the observational studies, the choice of both PPI and P2Y12 inhibitors was left to individual physician discretion.

A new meta-analysis reviewing RCTs of DAPT following PCI showed that DAPT trials after PCI often overlook gastroprotection entirely [65]. Among 21 trials, none incorporated any protocol for PPI or gastroprotection. However, five trials reported rates of PPI use (25.6-69.1%). Underutilization of gastroprotection was also reported in another study [66]. Trials utilizing DAPT in the future could provide clear guidelines on PPI use and strengthen the power of meta-analyses.

## Conclusions

Guidelines vary internationally recommending the concurrent use of PPI and DAPT. The 2010 ACCF/ACG/AHA guidelines recommend that only patients on DAPT with increased risk of UGIB should take PPI, while other guidelines call for gastroprotection for all patients on DAPT. Both aspirin and clopidogrel can interact with PPI, potentially decreasing their antiplatelet effects. However, the evidence for this is not particularly strong. COGENT was the only large RCT investigating patients on concurrent DAPT and PPI therapy. There have been smaller RCTs, various meta-analyses, and observational studies that have proposed conflicting evidence of this regimen’s efficacy. While some studies still warn of increased MACCE from concurrent therapy, other studies have shown beneficial effects such as a decrease in UGIB. Another large-scale RCT is needed to better understand the short- and long-term effects of concurrent DAPT and PPI use. Future studies should consider how dyspepsia symptoms affect treatment adherence, account for potential racial and ethnic differences, and account for baseline risk levels for related adverse events. Altogether, a more intensive study of DAPT/PPI co-prescription could provide patients with improved personalized care that meets their specific symptoms and accounts for their particular risk levels, with the goal of improving overall health outcomes.
